# Understanding the Epidemiology of Malaria in Zanzibar Through Molecular and Serological Analysis of Samples Collected During Reactive Case Detection

**DOI:** 10.1093/ofid/ofag051

**Published:** 2026-02-04

**Authors:** Varun Goel, Wahida Hassan, Caroline Murphy, Barbara B Choloi, Mohamed Ali, Bakari Mohamed, Abdallah Zacharia, Msolo C Dominick, Kyaw Thwai, Safia Mohammed, Shija J Shija, Jeffrey A Bailey, Anders Björkman, Billy E Ngasala, Eric Rogier, Jonathan J Juliano, Jessica T Lin

**Affiliations:** Department of Geography, University of South Carolina, Columbia, South Carolina, USA; Zanzibar Malaria Elimination Program (ZAMEP), Zanzibar, Tanzania; Department of Epidemiology, Gillings School of Global Public Health, University of North Carolina, Chapel Hill, North Carolina, USA; Division of Infectious Diseases, Department of Medicine, School of Medicine, University of North Carolina at Chapel Hill, Chapel Hill, North Carolina, USA; Zanzibar Malaria Elimination Program (ZAMEP), Zanzibar, Tanzania; Zanzibar Malaria Elimination Program (ZAMEP), Zanzibar, Tanzania; Department of Parasitology and Medical Entomology, Muhimbili University of Health and Allied Sciences, Dar es Salaam, Tanzania; Department of Parasitology and Medical Entomology, Muhimbili University of Health and Allied Sciences, Dar es Salaam, Tanzania; Division of Infectious Diseases, Department of Medicine, School of Medicine, University of North Carolina at Chapel Hill, Chapel Hill, North Carolina, USA; Zanzibar Malaria Elimination Program (ZAMEP), Zanzibar, Tanzania; Zanzibar Malaria Elimination Program (ZAMEP), Zanzibar, Tanzania; Department of Pathology and Laboratory Medicine, Brown University, Providence, Rhode Island, USA; Department of Global Public Health, Karolinska Institute, Stockholm, Sweden; Department of Parasitology and Medical Entomology, Muhimbili University of Health and Allied Sciences, Dar es Salaam, Tanzania; Department of Women's and Children's Health, International Maternal and Child Health (IMCH), Uppsala University, Uppsala, Sweden; Division of Digestive Disease and Nutrition, University of Kentucky, Lexington, Kentucky, USA; Department of Epidemiology, Gillings School of Global Public Health, University of North Carolina, Chapel Hill, North Carolina, USA; Division of Infectious Diseases, Department of Medicine, School of Medicine, University of North Carolina at Chapel Hill, Chapel Hill, North Carolina, USA; Curriculum in Genetics and Molecular Biology, University of North Carolina, Chapel Hill, North CarolinaUSA; Institute of Global Health and Infectious Diseases, University of North Carolina, Chapel Hill, North Carolina, USA; Division of Infectious Diseases, Department of Medicine, School of Medicine, University of North Carolina at Chapel Hill, Chapel Hill, North Carolina, USA; Institute of Global Health and Infectious Diseases, University of North Carolina, Chapel Hill, North Carolina, USA

**Keywords:** malaria, molecular epidemiology, serology, Tanzania, Zanzibar

## Abstract

**Background:**

The Zanzibar archipelago has been a pre-elimination region for malaria thanks to rigorous control interventions, but recent surges in malaria cases have been observed. The contribution of non-falciparum species to the current malaria situation is unknown.

**Method:**

This study investigates the epidemiology of falciparum and non-falciparum malaria species in Zanzibar. Leveraging plasma extracted from dried blood spots (DBS) collected during reactive case detection (RCD) activities across Unguja island from May 2022 to January 2023, we measured immunoglobulin G (IgG) responses to *Plasmodium* MSP1-19kD antigens using a multiplex bead-based immunoassay. Additionally, active infections were detected using species-specific real-time PCR.

**Results:**

Out of 1618 participants surveyed in 35 RCDs, 35.3% had exposure to any malaria species, with *Plasmodium falciparum* being the most common (29.8%). Seroprevalences for non-falciparum species were lower: *Plasmodium ovale* (5.8%), *Plasmodium malariae* (5.9%), and *Plasmodium vivax* (5.9%). Active infections were detected in 6.0% of participants, predominantly *P. falciparum* (4.6%). Travel to mainland Tanzania was a dominant risk factor for seropositivity for all 4 malaria species. Other factors associated with *Pf* seropositivity (high-risk occupations and female status) were not associated with seropositivity for non-falciparum species. The geographic distribution of non-falciparum exposure differed compared to falciparum, with relatively higher seroprevalences in rural districts, especially Kazkazini A in northern Unguja.

**Conclusions:**

This study suggests a significant contribution of non-falciparum species to the local epidemiology in Zanzibar. Current control and elimination efforts, focused on *P. falciparum*, may not adequately address exposure to non-falciparum species.

Zanzibar has been a pre-elimination region for over a decade due to the rigorous implementation of malaria control interventions including test and treat with artemisinin combination therapies (ACTs), reactive case detection (RCD), use of long-lasting insecticide-treated bednets, and vector control [1]. These interventions have reduced malaria prevalence to <1%, except during outbreaks that temporarily elevate case numbers [1]. However, there have been recent surges in malaria cases in the archipelago. In 2020, the annual parasite incidence for Unguja, the main island, reached 11.5 with 12 695 cases being reported [2]. Since then several outbreaks have occurred. Given that *Plasmodium falciparum* is responsible for the vast majority of morbidity and mortality associated with malaria, the majority of resources have gone into its control. However, in nearby mainland Tanzania, as *P. falciparum* has declined, transmission of non-falciparum malaria, in particular *Plasmodium ovale* and *Plasmodium malariae*, has persisted or even increased, potentially due to differences in how those species respond to malaria control interventions [3, 4]. Amidst high levels of connectivity and intensive control efforts that have reduced falciparum malaria prevalence in Zanzibar, we pursued a dedicated investigation of the epidemiology of non-falciparum malaria on the main island of Unguja.

Active malaria infection can be detected by multiple methods including microscopy, rapid diagnostic tests (RDTs) and nucleic acid testing; the most specific and sensitive of these being PCR [5–7]. This is particularly true for non-falciparum malaria, which relies on detection through the lactate dehydrogenase (LDH) band of RDTs, which has overall lower sensitivity than histidine-rich protein II (HRP2), which is specific for falciparum malaria [8]. However, in low-transmission settings, active infections may be so infrequent that PCR methods have limited utility. Serological assays can be used to assess prior exposure to malaria in a species-specific manner, thus giving a longer-term picture of malaria transmission and a better sense of exposure in low-transmission settings [9]. Over the last decade, there have been sporadic reports of active infection with *P. ovale* [10–13], *P. malariae* [10, 12–14], and *Plasmodium vivax* [12–14] in Zanzibar. However, there have been no serological surveys to reflect exposure over time of the various malaria species on the archipelago.

Asymptomatic infections that persist and are not treated provide a potential reservoir for malaria transmission [15]. Reactive case detections, typically using malaria RDTs to detect asymptomatic *P. falciparum* cases in the vicinity of an index case identified in a clinic, is often employed to find asymptomatic infections, but misses individuals with low-density infections. We leveraged dried blood spots (DBS) collected as part of RCD activities by the Zanzibar Malaria Elimination Program on the island of Unguja, to investigate previous exposure to *P. falciparum*, *P. malariae*, *P. ovale*, and *P. vivax* and compare it to the rates of active infection detected by real-time PCR in asymptomatic individuals. Risk factors for exposure to all 4 malaria species were further investigated.

## METHODS

### Study Design and Data Collection

We conducted 35 RCD visits among 50–100 nearest individuals to an index case across Unguja in Zanzibar between May 2022 and January 2023, spanning 2 rounds spaced 1 month apart, across rural and urban areas. A total of 1793 participants provided DBS, along with travel histories and detailed demographic and household information. The same individuals were not necessarily sampled at each visit, and individuals who were sampled twice were only included at the first sampling for analysis.

### Sample Processing

Three 6 mm punches were placed into a well of a 96-well deep-well plate for DNA extraction, which was performed using a Chelex–Tween extraction protocol [16]. A single 6 mm punch was placed in a separate deep-well plate for plasma extraction for serology, performed as previously described [17].

### Immunoglobulin G Assay Analysis and Threshold Selection

We used a multiplex bead-based immunoassay to detect immunoglobulin G (IgG) against *Plasmodium* MSP1-19kD antigens to measure exposure to *P. falciparum*, *P*. *malariae*, *P*. *ovale,* and *P. vivax* as previously described [17]. Thresholds of mean fluorescent intensity (MFI) at which an individual is considered seropositive for a particular malaria species were selected using the 2-component finite-mixture model (FMM) method [9, 17, 18]. The FMM method has been applied to quantify malaria exposure in various settings including Tanzania. The FMM method utilizes maximum likelihood estimation approaches to determine 2 unweighted subpopulations (components) through calculation of their means and variance, yielding 2 separate distributions for putative seropositive and seronegative individuals for each IgG target. Specifically, the threshold for the analysis was chosen by calculating the exp(lognormal mean + 3 * standard deviations) of the MSP1-19kD9kD antigen values, similar to the analysis by Rogier et al [9].

### Real-time PCR Detection of Malaria Species

*Plasmodium falciparum* parasitemia was determined using a quantitative real-time PCR targeting *varATS* as previously described [19, 20]. For *P. ovale*, *P. malariae*, and *P. vivax* detection, we conducted separate real-time PCR (qPCR) assays for each species, targeting the 18S ribosomal RNA (18S rRNA) gene, enabling both species detection and a semiquantitative assessment of parasitemia using a standard curve of diluted plasmids for parasitemia estimation (MRA-178, 179, and 180, BEI Resources, Manassas, VA) [16, 21]. A genome equivalent was based on a conversion factor of 6 plasmid copies per genome. All plates included negative controls.

### Statistical Analysis

We identified associations between potential geospatial, demographic, and behavioral factors, and previous exposure to falciparum and non-falciparum malaria, by fitting robust Poisson regression models using generalized estimating equations (GEEs), adjusting for individual age and clustering within the same households. Generalized estimating equation models and FMM estimations were implemented using “geepack” [22] and “mixR” package in R version 4.2.2 [23]. We also conducted sensitivity analyses to assess whether effect estimates and results are affected by the choice of thresholds based on the FMM method, and whether there may be potential cross-reactivity in IgG responses among the 4 species-specific *Plasmodium* antigens. Detailed description of the sensitivity analysis and results are provided in the supplement.

## RESULTS

### Study Population

Out of the 1793 samples collected in the context of 2 consecutive rounds of RCD in the index household and surrounding households, 4 (0.2%) were excluded from the analysis due to missing MSP1-19kD IgG data, and 171 (9.5%) were removed as they were associated with participants who had previously participated in the first RCD round. In total, samples from 1618 participants who participated in 35 RCD rounds from May 2022 to January 2023 were included in the final seroepidemiology analytical dataset. The PCR cohort represented a subset of those included in the seroepidemiology analysis, with 928/1618 (57%) participants sampled from May 2022 to September 2022 also tested for both active falciparum and non-falciparum infections using qPCR.

Overall, there were no notable differences between participants included in the seroepidemiology and qPCR cohorts, although the proportion of qPCR participants in urban settlements was higher compared to the seroepidemiology cohort (Table 1). For the seroepidemiology cohort, the median (interquartile range [IQR]) age was 15 (8–29) years, with more female (59%) participants. Most participants lived in urban settlements (72%) in Magharibi B, Magharibi A, and Mjini. Participants mainly consisted of students (44%), followed by housewives (14%), children (13%), and those engaged in trade or business (11%). Only 2.7% of participants reported traveling outside of Zanzibar to mainland Tanzania in the last 28 days. Additionally, 30% of participants reported having no bednets, while 59% reported not sleeping under a bednet the previous night. Twenty-four percent of participants reported indoor residual spraying (IRS) with 11% reporting last IRS within 6 months. Most participants (88%) reported living in houses made of brick, cement, or both, and almost all participants (98%) lived under a sheet metal, tin, or tiled roof.

**Table 1.** Baseline Characteristics of Participants in the Seroprevalence and qPCR Cohorts

Variable	Seroprevalence		qPCR	
…	N	(%)	N	(%)
Age	1618	…	927	…
<8	344	(21.3)	203	(21.9)
8–15	469	(29.0)	263	(28.4)
16–30	436	(26.9)	246	(26.5)
>30	369	(22.8)	215	(23.2)
Sex	1618	…	927	…
Female	957	(59.1)	533	(57.5)
Male	661	(40.9)	394	(42.5)
Settlement type	1618	…	927	…
Urban	1171	(72.4)	802	(86.5)
Rural	447	(27.6)	125	(13.5)
District	1618	…	927	…
Magharibi B (urban and rural)	495	(30.6)	311	(33.5)
Magharibi A (urban)	380	(23.5)	265	(28.6)
Mjini (urban)	380	(23.5)	226	(24.4)
Kati (rural)	180	(11.1)	42	(4.5)
Kusini (rural)	99	(6.1)	48	(5.2)
Kaskazini A (rural)	84	(5.2)	35	(3.8)
Occupation	1608	…	912	…
Student	704	(43.8)	421	(45.8)
Housewife	225	(14.0)	119	(12.9)
Child	216	(13.4)	113	(12.3)
Trader/business	179	(11.1)	99	(10.8)
Farming	73	(4.5)	35	(3.8)
Fishing	66	(4.1)	45	(4.9)
Teacher	23	(1.4)	13	(1.4)
Retired or unemployed	19	(1.2)	12	(1.3)
Public servant/NGO	16	(1.0)	8	(0.9)
Food service	11	(0.7)	6	(0.7)
Watchman	10	(0.6)	2	(0.2)
Construction	8	(0.5)	0	(0.0)
Tourism	6	(0.4)	2	(0.2)
Other	52	(3.2)	37	(3.9)
Number of bednets in home	1618	…	927	…
0	487	(30.1)	316	(34.1)
1–2	469	(29.0)	281	(30.3)
3+	662	(40.9)	330	(35.6)
Age of bednets	1107	…	612	…
<1 y	639	(57.7)	372	(60.8)
1–2 y	426	(38.5)	214	(35.0)
3+ y	42	(3.8)	26	(4.2)
Slept under bednet last night?	1099	…	600	…
Yes	624	(56.8)	352	(58.7)
No	475	(43.2)	248	(41.3)
Indoor residual spraying	1559	…	911	…
Yes	369	(23.7)	259	(28.4)
No	1190	(76.3)	652	(71.6)
Last indoor residual spraying	353	…	249	…
<6 m	40	(11.3)	38	(15.3)
6 to <12 m	280	(79.3)	190	(76.3)
>12 m	33	(9.3)	21	(8.4)
House material	1577	…	916	…
Bricks and cement	767	(48.6)	501	(54.7)
Cement	483	(30.6)	253	(27.6)
Bricks	136	(8.6)	50	(5.5)
Stone with mud	58	(3.7)	41	(4.4)
Mud	13	(0.1)	10	(1.1)
Bamboo with mud	11	(0.1)	11	(1.2)
Other (combination)	109	(6.9)	50	(5.5)
Roof	1479	…	864	…
Sheet metal/tin/tiles	1455	(98.4)	848	(98.2)
Thatched/leaves/bamboo	24	(1.6)	16	(1.8)
Water source within 50 m of house?	1512	…	903	…
Yes	189	(12.5)	113	(12.5)
No	1251	(82.7)	748	(82.8)
Don’t know	72	(4.8)	42	(4.7)
Travel	1540	…	919	…
None	1498	(97.3)	911	(99.1)
Mainland Tanzania	42	(2.7)	8	(0.9)

### Seroprevalence Estimates

**Figure 1.**
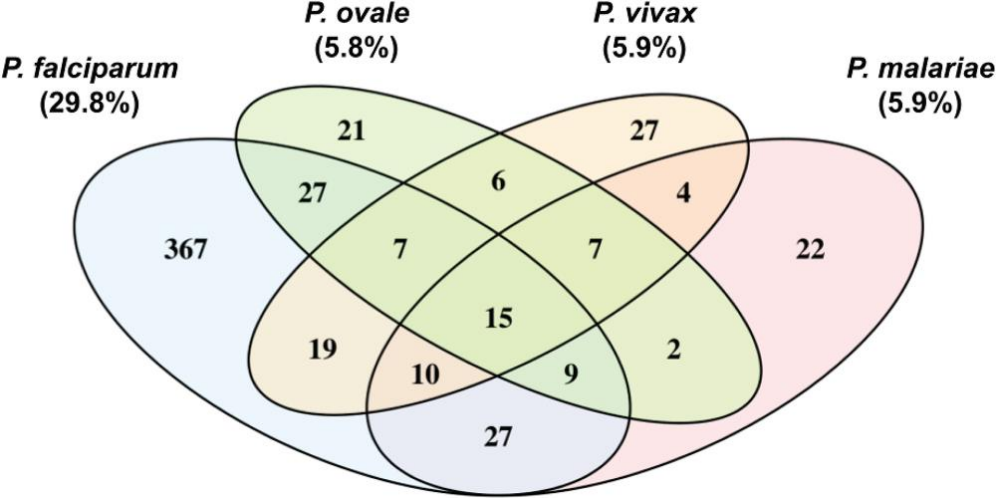
Overlap of seroprevalence across all 4 malaria species. In total, immunoglobulin G (IgG) versus MSP1-19kD responses were measured across 1618 Zanzibar residents participants in reactive case detection after a malaria case was identified in proximity (in their or a neighboring household). Note: Percentages for *Plasmodium ovale* were calculated for 1611 individuals with successful seroprevalence results. Percentages for other species were calculated for 1617 individuals. A total of 1611 participants had seroprevalence results available for all 4 species.

Out of the 1618 participants tested for anti-MSP1-19kD IgG antibody responses, 35% (n = 571/1617) had evidence for exposure to any malaria species. The most common exposure was *P. falciparum* with 30% of participants (n = 482/1617) being seropositive. Seroprevalence for each non-falciparum malaria species was much lower; *P. ovale* was 5.8% (n = 94/1611), while *P. malariae* and *P. vivax* seroprevalences were 5.9% (n = 96/1617 and n = 95/1617) each (Figure 1). Among participants with seroprevalence results available for all 4 malaria species (n = 1611), exposure to multiple malaria species was common with 133 (23% out of 570) having exposure to 2 or more species, and 15 (2.8%) exposed to all 4 species. Overall, 36% (203/570) of the malaria-exposed group demonstrated exposure to non-falciparum species; nearly half of these (44%, 89/203) showed exposure to non-falciparum species in the absence of exposure to falciparum.

We conducted sensitivity analysis by adopting a very stringent threshold at 5SD instead of 3SD, obtaining seroprevalence estimates for *P. falciparum*, *P. ovale*, *P. malariae*, and *P. vivax* of 24%, 2.4%, 3.0%, and 2.0%, respectively (Supplementary Tables 1 and 2). In this analysis, 23% (100/429) of the malaria-exposed group demonstrated exposure to non-falciparum species; nearly half of these (42%, 42/100) showed exposure to non-falciparum species in the absence of exposure to falciparum. To assess for potential cross-reactivity responses across antigens, we also examined correlation of MFI responses across all 4 MSP1-19kD orthologous antigens (Supplementary Figure 1), and the distributions in MFI responses for individuals with mono-species versus mixed-species exposure (Supplementary Figure 2). Although participants seropositive against multiple species had slightly higher MFIs to *Po* and *Pm* MSP1-19kD than those seropositive against only 1 species-specific antigen, those differences were not significant, even when assuming a 5SD threshold (Supplementary Figure 3).

**Figure 2.**
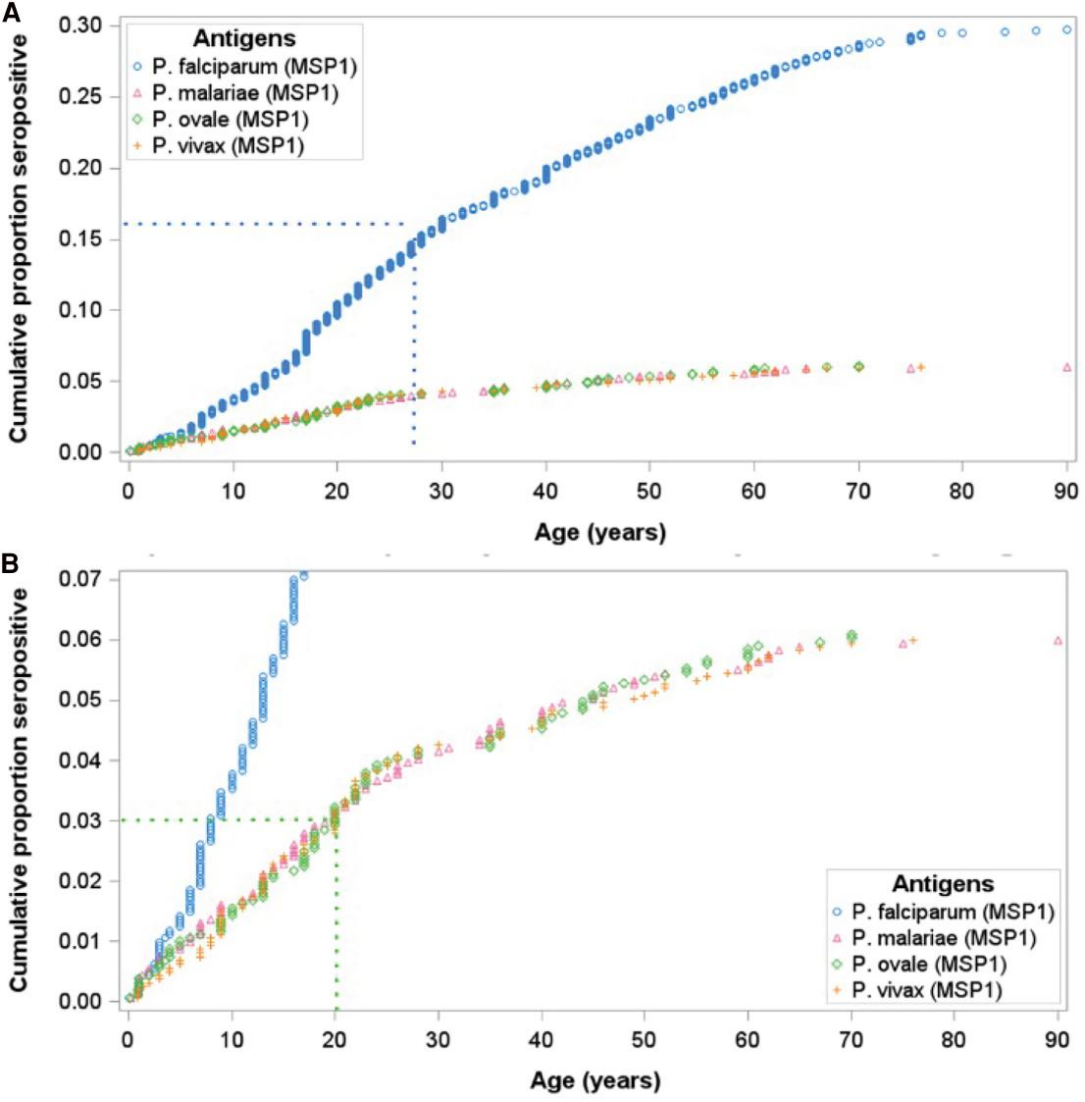
Cumulative exposure to malaria. (*A*) Overall cumulative exposure to all 4 malaria species tested. (*B*) Limited view at lower proportions to assess non-falciparum cumulative exposure.

We also used our cross-sectional data to estimate cumulative proportion of exposure over the lifespan to understand when exposure is usually acquired (Figure 2). Overall, approximately 30% participants were exposed to *P. falciparum*, with a steady increase in the proportion of participants seropositive over childhood years. By age 27 years, approximately 15% (half the total seroprevalence) of individuals demonstrated exposure. For non-falciparum infection, the rate of acquisition was similar between species and perhaps slightly faster than for *P. falciparum*, with half of those (3%) exposed by the age of 20 years.

### PCR Testing and Active Infections

Compared to seroprevalence estimates, rates of active infection were much lower. Overall, 56/928 (6.0%) were infected with any malaria species. Forty-three (4.6%) tested positive for *P. falciparum* parasitemia, with 77% of those participants (n = 33) having very low parasite density of <1 parasite/μL (Supplementary Figure 4). Similarly, few active non-falciparum infections were detected; *P*. *malariae* parasitemia was detected in 11 (1.2%), *P*. *ovale* in 4 (0.4%), and *P. vivax* in only 2 (0.2%) participants. Among these cases, only 2 reported recent mainland travel, to the Simayu region, Lake Zone (*P. malariae* case), and to Dar es Salaam (*P. ovale* case). Both *P. vivax* parasitemias were detected in the same household, in a housewife and a 4-month-old infant, both Zanzibari residents without recent travel.

### Factors Associated With Seropositivity

**Figure 3.**
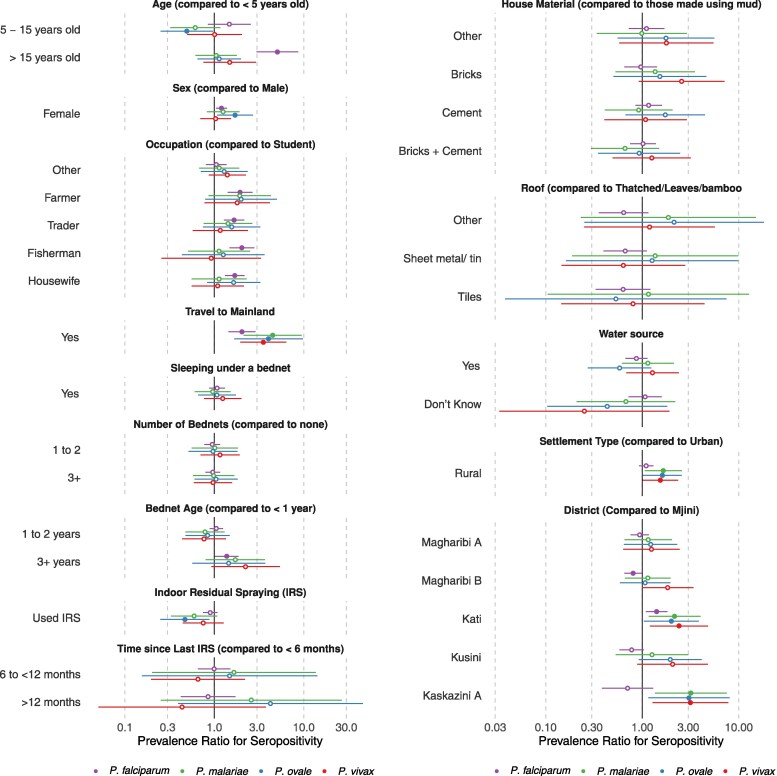
Factors associated with seropositivity for all 4 species. Seroprevalence ratios were estimated by fitting robust Poisson regression models using generalized estimating equations (GEEs) to each malaria species, adjusting for individual age and clustering of individuals within the same households. Error bars denote 95% confidence intervals. Filled point estimates denote results with *P* < .05.

Given low active infection rates by qPCR, especially for non-falciparum species, we only examined potential factors associated with prior malaria exposure. In this exploratory analysis, demographic factors associated with seropositivity showed differing trends for falciparum versus non-falciparum malaria (Figure 3; Supplementary Table 3). For *P. falciparum*, seroprevalence among persons greater than 15 years of age was 5.1 (95% CI: 3.0–8.7) times compared to under-5 children. In contrast, for *P. ovale*, children between 5 and 15 years of age had half the seroprevalence (PR = 0.49, 95% CI: 0.25–0.97) of *P. ovale* compared to under-5 children, with a similar trend for *P. malariae* (PR = 0.61, 95% CI: 0.32–1.16). Females, compared to males, had higher seroprevalence ratios for *P. falciparum* (PR = 1.2, 95% CI: 1.1–1.4) and *P. ovale* (PR = 1.7, 95% CI: 1.1–2.7) while controlling for age and clustering of individuals within households. Compared to students, occupations such as farming, trading, and fishing or being a housewife were associated with higher seroprevalence for *P. falciparum*, but not for any non-falciparum species. Associations among demographic factors and non-falciparum malaria were not significant after assuming a 5SD seropositivity threshold (Supplementary Figure 5).

Overall, travel to mainland Tanzania in the last 28 days was the factor most strongly associated with high seropositivity for all 4 species. Mainland travel was associated with twice the seroprevalence for *P. falciparum* (PR = 2.0; 95% CI: 1.4–2.9), and upwards of 3 times the seroprevalence for all non-falciparum malaria species (Figure 3). Sleeping under a bednet the previous night or number of household bednets was not associated with seropositivity for both falciparum and non-falciparum species. Bednets older than 3 years, however, were associated with higher *P. falciparum* exposure (PR = 1.37; 95% CI: 1.01–1.87) compared to less than a year-old bednets. Indoor residual spraying was associated with a protective effect only against *P. ovale* seroprevalence (PR = 0.47, 95% CI: 0.25–0.88). Household construction materials, roof type, and water source were not associated with seropositivity for any species. In our sensitivity analysis assuming a 5SD threshold, these associations persisted, with the exception of the association between IRS and *P. ovale* seroprevalence (Supplementary Figure 5).

### Geographic Distribution of Malaria Exposure

**Figure 4.**
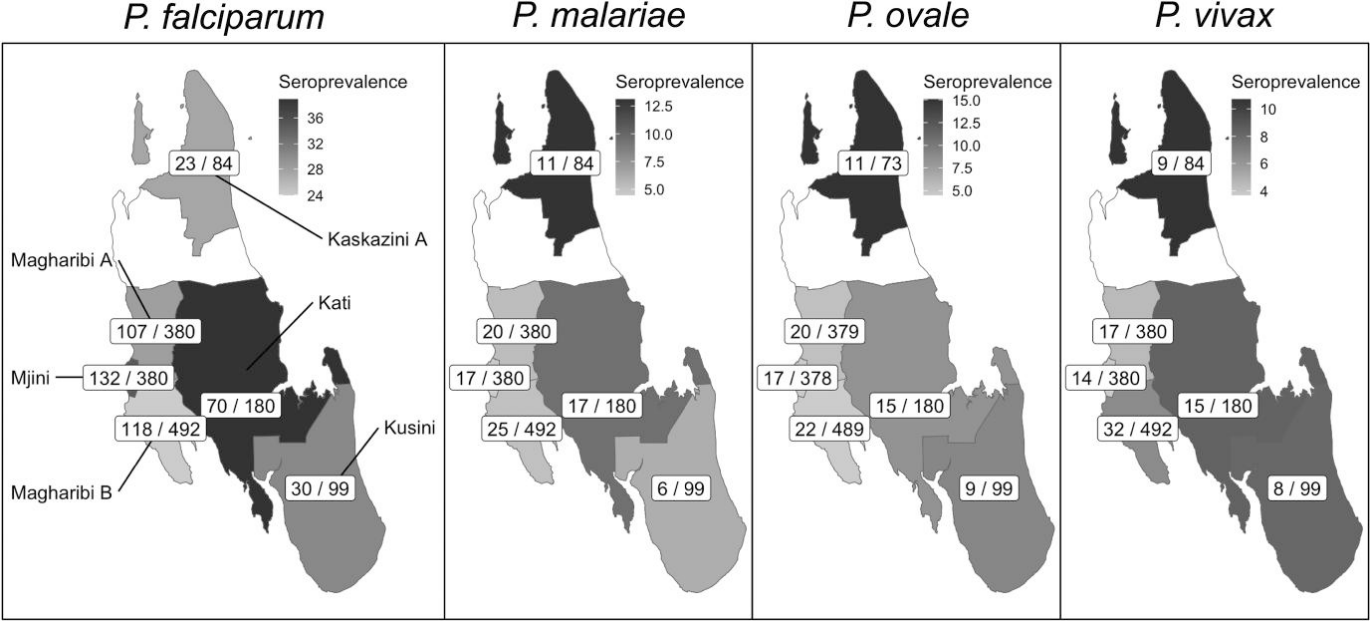
Map of geographic distribution of seroprevalence across all 4 species. Each district is shaded based on the species specific seroprevalence. Within each district is the number of seropositive samples (# positive/# tested).

We observed geographic differences in seroprevalence across Unguja for falciparum and non-falciparum malaria. Rural areas were associated with higher seroprevalence for all 3 non-falciparum species, but not with *P. falciparum* (Figure 3). Additionally, districts with the highest *P. falciparum* seroprevalence were not the same as districts with the highest seroprevalence for each non-falciparum species (Figure 4). The highest *P. falciparum* seroprevalence was found in the central district of Kati followed by the western district of Mjini, while higher non-falciparum seroprevalence (*P. malariae*, *P. ovale*, and *P. vivax*) was concentrated in Kaskazini A district in northern Unguja. Models controlling for age and household clustering also show that when compared to an urban district such as Mjini, non-falciparum seroprevalence ratios were significantly higher for rural districts such as Kati and Kaskazini A, but not other urban districts. Even with a 5SD seroprevalence threshold, the positive associations between non-falciparum seroprevalence and rurality persist, especially for *P. malariae*, although 95% CIs crossed the null for other non-falciparum species (Supplementary Figure 5). However, we also note that overall *P. falciparum* seroprevalence within the districts of Unguja is much higher (ranging from 25% to 35%), compared to non-falciparum species (ranging from 6% to 14%).

## DISCUSSION

We conducted a comprehensive epidemiological study of the four human malaria species in Zanzibar and compared exposure to active infection detected by qPCR in asymptomatic individuals. After nearly two decades of maintaining pre-elimination status, exposure to *P. falciparum* remains common (30%), perhaps attesting to both the longevity of IgG responses and ongoing malaria exposure, whether on Zanzibar or elsewhere. The longevity of anti-MSP1-19kD IgG responses varies by age and previous exposure as well as the type of stimulus, but MSP1-19kD is known to be a highly immunogenic malaria antigen with durable and detectable IgG levels in plasma for months to years, possibly decades [24–27]. Cumulative prevalence of exposure increased steadily with age, with about 50% of exposure occurring in the first three decades of life. Serological tools to measure (possibly distant) previous malaria exposure are especially important in low-transmission settings—as highlighted by the low rates of PCR-detected active infection in this study with only 4.6% of individuals infected with *P. falciparum*, mostly at very low density, under <1 parasite/μL blood.

Exposure to non-falciparum infection was lower but non-negligible and similar between the three species tested, at approximately 6%. Cumulative exposure for these species reached its midpoint slightly earlier, around 20 years of age. Though active infections were uncommon, they were more prevalent than previously seen in a large PCR survey conducted 6 years earlier, in 2016 [13]. Though only 2.7% of the study participants reported traveling outside of Zanzibar to mainland Tanzania in the last 28 days, exposure to non-falciparum infection was significantly higher in travelers compared to those not reporting recent travel. Although we did not collect data on more remote travel, *Plasmodium malariae*, *P. ovale*, and *P. vivax* all have biological mechanisms that can lead to persistent or chronic latent infection that may support maintenance of detectable IgG levels even if exposure was more remote.

Similarity of seroprevalence among non-falciparum species might raise concerns about cross-reactivity of IgG responses to orthologous MSP1-19kD antigens. Several of our analyses support that these signals represent true exposure. In this study, MFI intensity did not seem to vary with co-infection. Cross-reactivity between antibodies generated by a falciparum exposure and a non-falciparum antigen would likely present as lower non-falciparum MFI in the co-infections. Also, no specific patterns of correlation were determined. These antigens have been used extensively for seroepidemiology studies, and the risk of cross-reactivity has been studied and deemed to be low [18]. Given this, the reported seroprevalence is likely to be representative of exposure in this population. Applying more stringent cutoffs for seropositivity still yielded evidence of exposure to each non-falciparum species in the study population.

The significantly higher seroprevalence for *P. falciparum* among those older than 15 years of age may be explained by the fact that they had been exposed to falciparum before intensified malaria control and elimination in 2007–2008 in Zanzibar [1, 28]. While some seropositivity may have persisted, re-exposure in more recent years might have made the immune responses more significant, durable, and thus, detectable [24, 25]. In contrast, seropositivity to non-falciparum malaria was not significantly higher in adults compared to children, which may reflect relatively higher exposure to these parasites in more recent years. Interestingly, *P. ovale* and *P. malariae* seropositivity appeared higher in younger under-5 children than older children (5–15 years). This may also suggest a recent exposure in the population. Besides local exposure in rural areas, younger children are likely exposed when traveling with their parents to the mainland [29]. Potential reasons for a new relative emergence of non-falciparum malaria infections may be that they are not as readily diagnosed and treated. They cause less symptoms and are less detectable by mRDTs, leading to cryptic transmission, especially in the absence of *P falciparum* infections [30]. *Plasmodium ovale* and *P vivax* also require primaquine treatment of liver stages and are therefore not radically treated by ACTs only.

The relative geographic distribution of exposure to different malaria species was not the same. *Plasmodium falciparum* exposure was highest in Kati district in central-east Unguja. Kati has had relatively high annual parasite incidence (API) in the years leading into this study, with an API of 18.3 in 2020 and 11.6 in 2021 [2]. In 2022, the API was lower (5.3), which may in part be reflected in the infrequently detected active infections by qPCR during the study (43/928). The seroprevalence of all three non-falciparum species was highest in Kaskazini A, a lower-transmission area with APIs of 7.6, 3.3, and 1.7 in 2020, 2021, and 2022, respectively [2]. This is not surprising, as studies of active infection by non-falciparum malaria in Tanzania have shown elevated risks in lower-transmission regions [3, 4, 16].

Limitations to this study include its cross-sectional nature and possible bias toward increased malaria exposure compared to the general population, based on its RCD study design. This sampling strategy targets at-home asymptomatic persons during visits by the malaria surveillance officer. Therefore, it does not address the epidemiology of symptomatic malaria and likely under-samples populations engaged in occupational activities outside home. Our study was not large enough to perform fully adjusted multivariate analyses for factors associated with seropositivity. While the longevity of anti-MSP1-19kD IgG responses has been studied for *P. falciparum*, less is known about the longevity of response to the orthologous antigen in non-falciparum species. Additionally, despite the increased sensitivity of PCR for detecting non-falciparum species, the assays used likely still miss the lowest-density parasitemias that can be found in these species. Finally, given the possibility of persistent, latent, and relapsed infection, we are unable to determine whether the few active parasitemias of non-falciparum species were acquired locally or not.

Despite relatively low transmission, exposure to malaria, in particular falciparum malaria, remains high in Zanzibar with over one-third of the population being seroreactive to any malaria species. Immediately prior to data collection, there was a major surge of malaria in 2021, potentially highlighted by the high seroprevalence despite lower levels of active infection during 2022–2023. Seroreactivity to non-falciparum infections was also prevalent, including to *P. vivax*, which has historically been low in Africa [31]. Our data suggest that these species appear to have become relatively more prevalent and epidemiologically more important in recent years in Zanzibar. Further research is needed to better understand the context of exposure to non-falciparum malaria species. Questions remain over whether non-falciparum infections are escaping being adequately treated and/or whether they are escaping present vector control measures. Beyond targeting travelers, it is unclear whether current elimination efforts focused on *P. falciparum* will adequately address exposure to non-falciparum species.

## Supplementary Material

ofag051_Supplementary_Data

## Supplementary Data

Supplementary materials are available at Open Forum Infectious Diseases online. Consisting of data provided by the authors to benefit the reader, the posted materials are not copyedited and are the sole responsibility of the authors, so questions or comments should be addressed to the corresponding author.
